# Effect of Maternal Age on Pregnancy Outcome and Cesarean Delivery Rate

**DOI:** 10.14740/jocmr1904w

**Published:** 2014-11-19

**Authors:** Ali Ramazan Benli, Neriman Cetin Benli, Abdullah Taner Usta, Tolga Atakul, Mustafa Koroglu

**Affiliations:** a Department of Family Medicine, Faculty of Medicine, Karabuk University, Karabuk, Turkey; b Baglar ASM, Safranbolu, Karabuk, Turkey; c Department of Gynecology and Obstetrics, Bagcilar Education and Research Hospital, Istanbul, Turkey; d Department of Gynecology and Obstetrics, Karabuk Education and Research Hospital, Karabuk, Turkey; e Department of Hematology, Karabuk Education and Research Hospital, Karabuk, Turkey

**Keywords:** Advanced maternal age, Obstetric outcomes, Cesarean delivery

## Abstract

**Background:**

The aims of this retrospective study were to evaluate the maternal and prenatal outcomes between 35 years and older pregnancies and younger pregnancies, and the effects of the age of pregnancy, mother and newborn.

**Methods:**

Pregnant women who gave birth in Vakif Gureba Training and Research Hospital, Clinic of Obstetrics and Gynecology in 2006 were retrospectively screened. Pregnant women aged 35 years and over were included in this study and the pregnant women between age range of 30 - 34 years were included in the control group.

**Results:**

Pregnancy rate was found as 7.1% in 35 years and older women in all the deliveries, cesarean delivery rate was found as 46.1% in this group at 1 year period. However, cesarean delivery rate was 40.9% in the control group. Cesarean delivery rate was found as 31.6% in all the deliveries. The most common cause of cesarean section indication was fetal distress in advanced maternal age (AMA) (11.7%), whereas previous cesarean section was found as the most common cause in the control group (15.1%).

**Conclusion:**

No significant difference was found between AMA group and normal pregnancies in terms of preterm labor, caesarian section, morbidity, mortality and chronic diseases such as hypertension and diabetes mellitus.

## Introduction

Advanced maternal age (AMA) is defined as age ≥ 35 years old at the time of delivery and is considered to be among the risky pregnancies. As of 2009, 14% of all the children were born by 35 years and older mothers [1]. Pregnancies of high risk have increased mortality and morbidity for both mother and fetus during birth [2]. Several conditions such as stress, fatigue, posture of working, sight working, early or AMA, exposure to chemicals and socioeconomic status lead to an additional burden on working pregnant women [3]. Today, prolonged education, career priority, fertility control through effective contraceptive methods, heavy working conditions and economic problems lead to postponed pregnancy age [4, 5].

In this study, we investigated and compared maternal, perinatal and newborn outcomes of pregnancy age of younger and older than 35 years.

## Material and Methods

All pregnant women who gave birth in 2006 in the Department of Obstetrics and Gynecology of Vakif Gureba Training and Research Hospital, were retrospectively investigated. Pregnant women aged 35 years and older were grouped for study group (group 1) and between ages of 30 and 34 years (group 2) were included as a control group.

Parameters which are compared between study and control groups are as follows: perinatal and obstetric complications, maternal and fetal mortality and morbidity rates, Apgar scores, gestational diabetes mellitus, early membrane rupture, preterm birth, intrauterine growth retardation (IUGR), delivery method, frequency of newborn anomalies, presence of eclampsia and preeclampsia, frequency of additional diseases, birth weight, hemoglobin values and smoking status.

### Statistical analysis

Data from this study were analyzed using SPSS for Windows version 16.0 software. Student’s *t*, Fisher exact, Mann-Whitney U and Chi-square tests were used for comparisons of study and control groups’ data. P values < 0.05 were considered statistically significant.

## Results

Of the 2,162 pregnant women who presented to our clinic, 154 (7.12%) were ≥ 35 years old, while 298 (13.7%) were in the age range of 30 - 34 years.

The pregnancy rate of women aged ≥ 35 years was found to be 7.12%. The cesarean delivery rate in this group was 46.1% whereas the cesarean delivery rate was 40.9% in the control group. The cesarean delivery rate was found to be 31.6% in all the deliveries (Table 1). The most common indication for cesarean delivery was fetal distress in the AMA group (11.7%), whereas the most common indication in the control group (15.1%) was a previous cesarean delivery (Table 2). Ratio of gestational diabetes mellitus (GDM) was 6.5% in the study group and was 3.4% in the control group, although this difference was not statistically significant (Table 3) (P > 0.05). No statistically significance difference was observed in the incidence of early membrane rupture (EMR), preterm birth and IUGR (Table 3) (P > 0.05). No statistical significance was found between the study and control groups in terms of mortality and anemia (Table 4, 5) (P > 0.05).

**Table 1:** Cesarean Delivery Rates

	30 - 34 years old	≥ 35 years old	All deliveries
Cesarean delivery rate	40.90%	46.10%	31.60%

**Table 2:** Rates of Cesarean Delivery Indications

Indication for cesarean delivery	30 - 34 years old	≥ 35 years old
Fetal distress	5.4	11.7
Previous cesarean delivery	15.1	10.4
Cephalopelvic disproportion	2.7	0
Malpresentation	4.0	7.1
Labor dystocia	3.4	3.2
Placenta previa	2.0	2.6
Abruptio placentae	0.7	3.2
Vasa praevia	1.0	0
Large birth weight	1.7	1.3
Precious baby	2.3	1.3
Oligohydramnios	3.4	3.9
Other	2.7	4.5

**Table 3:** Rates of GDM, EMR, Preterm Labor and IUGR

Age	30 - 34 years		35 years and older		Chi-square	P
	n	%	n	%		
Gestational diabetes mellitus						
No	288	96.6	144	93.5		
Yes	10	3.4	10	6.5	2.36	0.124
Early membrane rupture						
No	290	97.3	153	99.4		
Yes	8	2.7	1	0.6		0.176
Preterm labor						
No	278	93.3	145	94.2		
Yes	20	6.7	9	5.8	0.12	0.721
Intrauterine growth retardation						
No	287	96.3	152	98.7		
Yes	11	3.7	2	1.3		0.235

**Table 4:** Perinatal Mortality Rates

Age	30 - 34 years		35 years and older		Chi-square	P
	n	%	n	%		
Mortality						
No	286	96.0	149	96.8		
Yes	12	4.0	4	2.6	0.60	0.436
Mortality yes						
Baby	12	4.0	4	2.6		
Mother	0	0	1	0.6		

**Table 5:** Rates of Congenital Anomalies

Age	30 - 34 years		35 years and older		P
	n	%	n	%	
Anomaly					
No	293	98.3	152	98.7	
Yes	5	1.7	2	1.3	0.554
Anomaly yes					
CNS anomaly	3	1.0	1	0.6	
Extremity	1	0.3	-	-	
Other	1	0.3	1	0.6	

CNS: central nervous system.

Since the normal values in the table were significant, mortality values were combined as “yes” or “no” and calculated again.

The incidence of preconceptual chronic diseases was 21.4% in the pregnant women aged ≥ 35 years and 16.1% in the controls, although this difference was not statistically significant (Table 6) (P > 0.05).

**Table 6:** Preconceptional Comorbidity and Preeclampsia Rates

Age	30 - 34 years		≥ 35 years old		Chi-square	P
	n	%	n	%		
Comorbidity						
No	250	83.9	121	78.6		
Yes	48	16.1	33	21.4	1.95	0.162
Comorbidity yes						
Diabetes mellitus	2	0.7	3	1.9		
Hypertension	3	1.0	8	5.2		
Asthma	13	4.4	5	3.2		
Cardiac	8	2.7	5	3.2		
Other	22	7.4	12	7.8		
Preeclampsia						
No	289	97.3	150	98.0		
Yes	8	2.7	3	2.0		0.756

Difference between study and control groups was not significant for preeclampsia rates (2.0% and 2.7% respectively; P = 0.756).

The numbers of gravida and parity were found to be significantly higher in study group, while no significant difference was found between groups in terms of abortion rates, gestational age, mean birth weight, anemia and the first minute Apgar scores (Table 7).

**Table 7:** Gravida, Parity, Abortions, Birth Weight of Newborn, Maternal Hemoglobin Values and First Minute Apgar Scores

Age	30 - 34 years		≥ 35 years old		P
	Mean	SD	Mean	SD	
Gravida	3.35	1.78	4.77	2.50	0
Parity	1.86	1.45	3.06	2.11	0
Abortions	0.49	0.91	0.68	1.03	0.055
Live born	1.78	1.35	2.87	1.88	0
Gestational age	38.36	3.34	38.21	3.35	0.649
Birth weight	3,204.73	709.72	3,201.49	734.47	0.964
Hemoglobin	11.52	1.563	11.319	1.411	0.181
Apgar scores	7.71	2.11	7.61	1.98	0.623

No statistically significant difference was found in terms of hemoglobin values studied in patients with anemia (Table 7) (P > 0.05).

## Discussion

Pregnancy is considered as a physiological and unique period in women’s life. However, an unpredictable disease of mother or fetus may complicate the pregnancy. Pregnancy is defined as “high-risk” if the possibility of an adverse outcome is higher than in the general population [6].

A high-risk pregnancy is a physiological, social and emotional condition, which threatens maternal and fetal health and increases the rates of mortality and morbidity [7]. Morbidity and mortality rates increase in antenatal, prenatal and postnatal periods in high-risk pregnancies compared to normal-risk pregnancies [2].

There are numerous studies about the effect of maternal age on pregnancy as a risk factor. Although recent studies have focused on the pregnancies of ≥ 35 years [8-10], there are several studies conducted, evaluating the pregnancies of women ≥ 40 years, even 45 years old as a standalone risk factor [5, 11]. Today, especially in the developed countries, many women postpone having children to the fourth decades of their lives due to social, economic and educational reasons. This condition takes place in an increasing manner day by day through global population [4]. Increasing birth rate of AMA (≥ 35 years) women in developed countries is a result of couples postponing marriage and child bearing [12-14]. Because of this changing trend to give birth, it is a contradictory question that who is under risk [15]. Despite numerous definitions for “elderly gravida”, this term is widely used for the women who have babies after the age of 35 [16]. However, some scientists who are working on 45 and higher aged women prefer to give definition of “very advanced maternity age” to women which are above 45 of age [12, 13].

Primigravid-infertile women constitute the biggest group of very advanced maternity aged women owing to advanced reproductive technologies. Modern infertility treatment methods, including oocyte donation and modern infertility treatment methods increase the number of women becoming pregnant in advanced ages [17-19]. These pregnancies are problematic since they have to cope with chronic disease of advanced age. Problems of these pregnancies include complications related to chronic diseases as well as AMA. Medical conditions like hypertension and diabetes are more common in women who become pregnant in advanced ages. Even healthy women aged ≥ 35 years have a higher risk of developing hypertension and diabetes during pregnancy [1]. Results of 154 pregnant women aged ≥ 35 were analyzed in our study group. A total of 298 pregnant women in the age range of 30 - 34 were included in our study as the control group. As it was expected, pregnancy (gravida) and the number of birth (parity) were found to be higher in the study group than in the control group due to difference of age. This difference was statistically significant (Table 7).

An increase was observed in the cesarean delivery rate, which is consistent with the literature about AMA [18, 19]. In the general population, the cesarean delivery rate was 31.6%, in pregnant women in the age range of 30 - 34, the rate was 40.9% and in pregnancies aged ≥ 35 years, the rate was 46.1% (Table 1). In a study by Hoque et al with 341 advanced maternal aged women, the rate of cesarean delivery was 38.4% [20]. In a meta-analysis by Smith et al with 297,842 women, in 2012 the rate of cesarean delivery was 25.7% in women ≥ 35 years old [10]. The most common indication of cesarean delivery was fetal distress in AMA (11.7%) and previous cesarean delivery (15.1%) in the control group (Table 2).

In our study, preconceptual comorbidities were found in 21.4% of the study and in 16.1% of the control group (Table 6). Increasing of diseases emerges in the older group due to several risk factors caused by aging process. Chronic hypertension was the most common comorbidity seen in study group, while asthma was the dominant comorbidity in control group. According to literature, it has been proposed that hypertension, and some other chronic diseases have been observed more with aging; however, perinatal complications could not be explained by this assumption [21].

Despite the literature which reported an increase in gestational diabetes with AMA, no significant difference was found in our study [5, 21-23] (Table 3).

In the published papers, preeclampsia was found to increase in the beginning and end of the reproductive period. In a retrospective study by Hoque et al, a statistically significant correlation was found between the AMA and low birth weight as a result of diabetes, preeclampsia and placenta previa [20]. On the contrary, in our study, in terms of preeclampsia no significant difference was observed in our study between the study and control groups.

There is not any evidence in the literature suggesting an increase in the rate of mortality in the pregnant women aged ≥ 35 years [24]. Similarly, we could not find any significance in terms of mortality (Table 4) (P > 0.05).

In the studies conducted, similar results were obtained for congenital malformations (major and minor anomalies) that were diagnosed antenatal in young and advanced aged patients [15]. Results of our study yielded similar outcomes (Table 5). Israel et al found anomalies by 10% related to karyotype in the fetuses of women aged over 45 years [25].

First minute Apgar scores were investigated to evaluate perinatal outcomes in newborns. According to the literature, lower Apgar scores were observed in the older pregnant women. In a study from China by Wen et al (2013), advanced age is correlated with low Apgar scores in pregnancies who delivered vaginally [26]. However, in a study by Dulitzki et al, it has been shown that the five minute Apgar score was not affected by maternal age [15]. In our study, no significant difference was found between the groups in terms of the first minute Apgar scores and birth weights (Table 7) (P > 0.05).

No significant difference was found between groups in terms of anemia based on the hemoglobin value during the hospitalization for delivery [6].

An important observation in our study was that 11.7% of the pregnant women were smoking during their pregnancies, and only 27.5% of the patients were regularly attending their prenatal visits. In a study by Lamminpaa et al in 2012, 10% of women were found to smoke during their pregnancies [27]. This demonstrated that better awareness of the society is needed about their controls by physicians.

### Conclusion

There is not any significant difference between pregnancies of advanced maternal aged patients and low-risk patients in terms of preterm birth, delivery method, morbidity, mortality and chronic diseases such as hypertension and diabetes mellitus. Although AMA carries certain risks, advances in especially oocyte donation techniques enable increases of the AMA rates. However, further studies with larger series of patients are needed to elucidate risks of AMA.
